# Chromosomal instability in Afrotheria: fragile sites, evolutionary breakpoints and phylogenetic inference from genome sequence assemblies

**DOI:** 10.1186/1471-2148-7-199

**Published:** 2007-10-24

**Authors:** Aurora Ruiz-Herrera, Terence J Robinson

**Affiliations:** 1 Evolutionary Genomics Group, Department of Botany & Zoology, University of Stellenbosch, Private Bag X1, Matieland 7602, South Africa; 2 Dipartimento di Genetica e Microbiologia, Universita' degli Studi di Pavia, Via Ferrata 1, Pavia 27100, Italy; 3 Evolutionary Genomics Group, Department of Botany & Zoology, University of Stellenbosch, Private Bag X1, Matieland 7602, South Africa

## Abstract

**Background:**

Extant placental mammals are divided into four major clades (Laurasiatheria, Supraprimates, Xenarthra and Afrotheria). Given that Afrotheria is generally thought to root the eutherian tree in phylogenetic analysis of large nuclear gene data sets, the study of the organization of the genomes of afrotherian species provides new insights into the dynamics of mammalian chromosomal evolution. Here we test if there are chromosomal bands with a high tendency to break and reorganize in Afrotheria, and by analyzing the expression of aphidicolin-induced common fragile sites in three afrotherian species, whether these are coincidental with recognized evolutionary breakpoints.

**Results:**

We described 29 fragile sites in the aardvark (OAF) genome, 27 in the golden mole (CAS), and 35 in the elephant-shrew (EED) genome. We show that fragile sites are conserved among afrotherian species and these are correlated with evolutionary breakpoints when compared to the human (HSA) genome. Inddition, by computationally scanning the newly released opossum (*Monodelphis domestica*) and chicken sequence assemblies for use as outgroups to Placentalia, we validate the HSA 3/21/5 chromosomal synteny as a rare genomic change that defines the monophyly of this ancient African clade of mammals. On the other hand, support for HSA 1/19p, which is also thought to underpin Afrotheria, is currently ambiguous.

**Conclusion:**

We provide evidence that (i) the evolutionary breakpoints that characterise human syntenies detected in the basal Afrotheria correspond at the chromosomal band level with fragile sites, (ii) that HSA 3p/21 was in the amniote ancestor (i.e., common to turtles, lepidosaurs, crocodilians, birds and mammals) and was subsequently disrupted in the lineage leading to marsupials. Its expansion to include HSA 5 in Afrotheria is unique and (iii) that its fragmentation to HSA 3p/21 + HSA 5/21 in elephant and manatee was due to a fission within HSA 21 that is probably shared by all Paenungulata.

## Background

Analyzing how mammalian genomes are organized and how chromosomal rearrangements are involved in speciation and macroevolution are fundamental to understanding the dynamics of mammalian chromosomal evolution. Phylogenetic analysis of both nuclear and mitochondrial DNA [1-6], among others, as well as the insertion sites of multiple long interspersed elements (LINE, [7]) and long terminal repeats (LTRs, [8]), all support the division of extant placental mammals into four major clades. These are Laurasiatheria and Supraprimates that together form Boreoeutheria (with a northern hemisphere origin), and Xenarthra and Afrotheria which have a Gondwanan (southern hemisphere) genesis, although this biogeographic hypothesis is not without detractors [9]. Although Afrotheria is usually depicted as basal in sequence based phylogenies, the first divergence in the placental tree has been a matter of concern for some time. For example, some argue [8] for a basal Xenarthra (the so-called Epitheria hypothesis, [10]) on insertion sites of retroposed elements (but see [11] for a contrary view), while data from 218 genes encompassing 205 kb of sequence resulted in a highly supported phylogeny that places the root between Afrotheria and other Placentalia [6], consistent with the basal Afrotheria or Exafroplacentalia hypothesis [4]. Most recently, however, Xenarthra and Afrotheria have been placed on a common basal branch (the Atlantogenata or Xenafrotheria hypothesis), to the exclusion of Boreoeutheria [12].

Afrotheria, the focus of our study, includes six mammalian orders all with an Afro-Arabian origin that exhibit extreme morphogical diversity and niche preference thought to result from the long period of isolation when Africa was an island continent 105-25 mya [13]. The six orders are Proboscidea (elephant), Sirenia (manatees and dugongs), Hyracoidea (hyrax), Tubulidentata (aardvark), Macroscelidea (elephant shrews) and Afrosoricida (golden moles and tenrecs). In cases where the analysis of primary sequences generates ambiguous phylogenetic results, "rare genomic changes" (RGCs, [14]) such as indels, LINEs, SINEs and chromosomal rearrangements have been widely viewed as markers that could, given their low levels of homoplasy, provide additional resolution to seemingly intractable phylogenetic problems (see [15] for application of SINEs in vertebrate phylogenetics). So far, afrotherian monophyly is supported by a suite of sequence-based characters that include a 9 bp deletion in exon 11 of the BRCA1 gene [16], 5' and 3' deletions in exon 26 of apolipoprotein B [5], the presence of a unique family of SINEs (AfroSINEs, [17-19]), long interspersed elements (LINE 1, [7]) and long terminal repeat (LTR) elements [8]. Consistent with the view that chromosomal rearrangements are similarly rarely homoplasious, and therefore robust indicators of evolutionary change (a default rate of 1–2 changes per 10 million years of mammalian evolution has been suggested, [20-22]), it comes as no surprise that in addition to providing evidence in support of the uniqueness of Afrotheria, chromosomal syntenies have also proved useful for clarifying the phylogenetic relationships within the group [23,24].

One of the most important features of mammalian chromosomal evolution is the suggestion of a non-random distribution of regions implicated in evolutionary chromosomal reorganization, the so-called "fragile-breakage" hypothesis [25]. Related to this, recent experimental data have demonstrated a correlation between the location of fragile sites and evolutionary breakpoints [26-28] suggesting that these unstable regions could be one of many factors implicated in the evolutionary process. At the cytogenetic level, fragile sites are expressed as non-stained gaps and breaks when cells are cultured under specific conditions [29]. In general, fragile sites can be expressed by agents such as aphidicolin, BrdU and 5-azacytidine among others, which delay or inhibit DNA replication or repair [30]. According to their frequency in the human population, as well as their mechanisms of expression, fragile sites have been conventionally classified into two groups: common and rare [31]. Common fragile sites in particular have been studied in different mammalian species confirming the initial hypothesis that they are structural characteristics of mammalian chromosomes [31]. Common fragile sites have been expressed in rodents [32-37], pig and cow [38-41], horse [42], cat [43-45], dog [46,47] and different primate species [26,48-51]. However, in all instances the species studied group within Laurasiatheria and Supraprimates, the most distant relatives of Xenarthra and Afrotheria that are thought to have diverged ~93 mya [52]. There is, at this point, no comparable data for the deeper divergences such as the Afrotheria whose separation from Boreoeutheria and Xenarthra is estimated at ~105 mya [52].

In an attempt to test if there are loci with a high tendency to break and reorganize in the Afrotheria, and whether these are coincidental with evolutionary breakpoints, we have analyzed the expression of aphidicolin-induced common fragile sites in fibroblast cultures from different specimens of three afrotherian species. These are the aardvark (*Orycteropus afer*, OAF, Tubulidentata), golden mole (*Chrysochloris asiatica*, CAS, Afrosoricida) and elephant-shrew (*Elephantulus edwardii*, EED, Macroscelidea). Given the position of Afrotheria near the root of Placentalia (species on the so-called "eutherian" side of the "metatherian-eutherian" dichotomy), the analysis of chromosomal instability in these species provides a unique opportunity to further our understanding of the mechanisms underpinning mammalian chromosomal evolution.

## Results

### Fragile site expression

#### (i) *Orycteropus afer*

A total of 652 metaphase spreads were analysed in two specimens, 374 from cultures treated with 0.2 μM APC, and 278 from control cultures (table 1). As expected, cells treated with aphidicolin presented the highest number of chromosomal aberrations (51.34% of the total metaphases), a 13-fold increase with respect to the control cultures. A total of 287 chromosomal abnormalities were detected of which the most common aberrations were chromatid breaks (61–94% of all aberrations detected, see table 2).

**Table 1:** Numbers of metaphase cells analysed, chromosomal breaks (gaps included) and chromatid breaks (gaps included) per metaphase observed in aphidicolin-treated and control cultures from the all afrotherian species and specimens studied.

	**0.2 μM APC**				**CONTROL**			
**Specimen**	**Metaphase cells analysed**	**Normal (%)**	**Aberrant (%)**	**Breaks/Metaphase**	**Metaphase cells analysed**	**Normal (%)**	**Aberrant (%)**	**Breaks/Metaphase**

**OAF1**	130	48 (36.92%)	82 (63.08%)	1.64	132	127 (96.21%)	5 (3.78%)	0.045
**OAF3**	244	134 (54.92%)	110 (46.08%)	0.59	146	140 (95.89%)	6 (4.11%)	0.041

*total*	*374*	*182 (48.66%)*	*192 (51.34%)*	*1.12*	*278*	*267 (96.04%)*	*11 (3.96%)*	*0.043*

**CAS1**	134	33 (24.66%)	101 (75.34%)	1.42	223	204 (91.48%)	19 (8.52%)	0.10
**CAS2**	165	51 (30.91%)	114 (69.09%)	1.60	209	197 (94.26%)	12 (5.74%)	0.05
**CAS3**	183	67 (36.61%)	116 (63.39%)	0.95	133	124 (93.23%)	9 (6.77%)	0.07

*total*	*482*	*151 (31.33%)*	*331 (68.67%)*	*0.99*	*565*	*525 (92.92%)*	*40 (7.08%)*	*0.07*

**EED1**	188	49 (26.06%)	139 (73.94%)	1.29	233	134 (90.54%)	14 (9.46%)	0.10
**EED2**	170	29 (17.06%)	141 (82.94%)	1.44	164	150 (89.82%)	17 (10.18%)	0.12
**EED3**	162	46 (28.40%)	116 (71.60%)	1.23	193	177 (91.71%)	16 (8.29%)	0.07
**EED4**	100	24 (24%)	76 (76%)	1.36	111	104 (93.69%)	7 (6.31%)	0.09

*total*	*620*	*148 (23.87%)*	*280 (78.21%)*	*1.33*	*619*	*565 (91.32%)*	*54 (8.77%)*	*0.09*

Abbreviations – OAF: *O. afer*, CAS: *C. asiatica *and EED: *E. edwardii*.

**Table 2:** Number of chromosomal aberrations observed in cells analysed from aphidicolin-treated cultures of three afrotherian species.

	**Chromosomal aberrations**				
**Specimens**	**chr breaks**	**chr gaps**	**cht breaks**	**cht gaps**	**total**

**OAF1**	8 (5.59%)	46 (32.17%)	87 (60.84%)	2 (1.40%)	143
**OAF3**	1 (0.69%)	5 (3.47%)	135 (93.75%)	3 (2.08%)	144

**CAS1**	1 (0.69%)	5 (3.47%)	135 (93.75%)	3 (2.08%)	144
**CAS2**	2 (1.10%)	10 (5.49%)	152 (83.52%)	18 (9.89%)	182
**CAS3**	3 (1.71%)	38 (21.71%)	124 (70.86%)	10 (5.71%)	175

**EED1**	0 (0%)	13 (5.35%)	224 (92.18%)	6 (2.47%)	243
**EED2**	6 (2.45%)	46 (18.78%)	183 (74.69%)	10 (4.08%)	245
**EED3**	0 (0%)	50 (25%)	135 (67.65%)	15 (7.5%)	200
**EED4**	1 (0.74%)	35 (25.74%)	92 (67.65%)	8 (5.88%)	136

Abbreviations – OAF: *O. afer*, CAS: *C. asiatica*, EED: *E. edwardii*, *chr breaks*: chromosome breaks, *chr gaps*: chromosome gaps, *cht breaks*: chromatid breaks and *cht gaps*: chromatid gaps.

**Figure 1:**
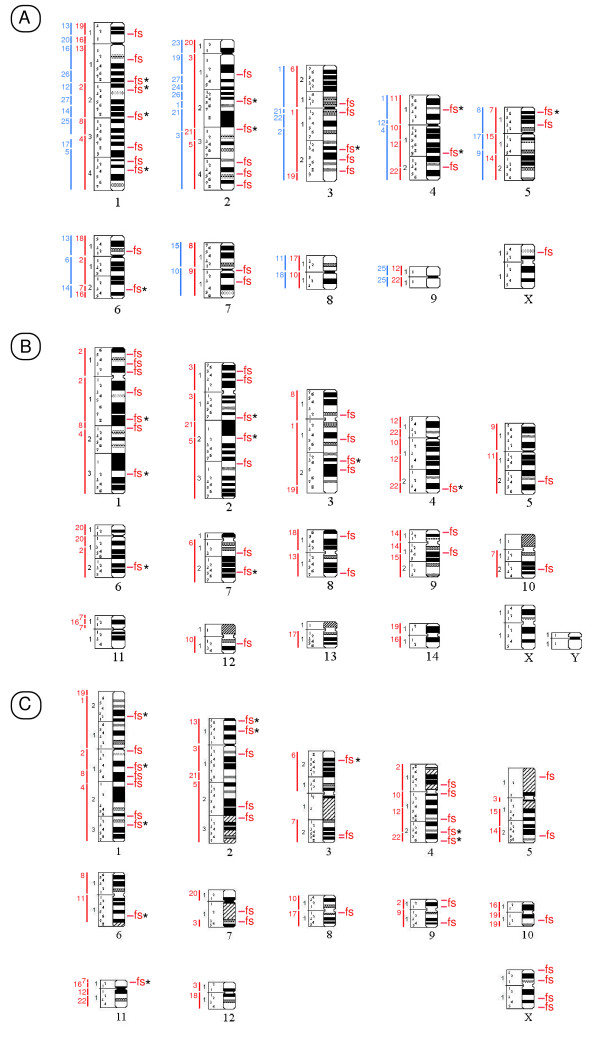
**Ideograms representative of *O. afer *(A), *C. asiatica *(B) and *E. edwardii *(C) chromosomes**. The regions of homology with human chromosomes [see 24, 53, 54] are depicted in red and indicated by the numbers to the left of each chromosomal schematic. In aardvark, homology with the African elephant chromosomes is shown in blue. The location of aphidicolin-induced fragile sites (fs) found in all specimens studied is indicated to the right of each chromosomal schematic in the three afrotherian species. Asterisks mark fragile sites conserved in at least two of the three species studied. Heterochromatic regions are marked by diagonal lines.

For each data set, the FSM program used in our analysis of fragile sites provides a critical value (C_α_) that indicates the minimum number of breaks needed for a chromosomal band to be considered fragile. This value was ≥3 for aardvark cultures treated with 0.2 μM aphidicolin. The number of fragile sites detected ranged from 23 in OAF specimen 1 to 12 in OAF specimen 2. Given the intraspecific variability of fragile site expression in mammalian species, we combined all expressed sites into a single species-specific analysis (this was also done for the golden mole and elephant shrew, see below). On this basis, a total of 29 sites were considered fragile in the aardvark (figure 1a and table 3) showing that there are regions in this species' genome that are prone to breakage under specific culture conditions; of these, six were expressed in both aardvark specimens (1p11, 1q18, 1q44, 2q13, 2q15 and 3p11, table 3).

**Table 3:** Chromosomal bands which are considered to contain fragile sites in the two aardvark (OAF) specimens studied.

	**No. aberrations**	
**band**	**OAF 1**	**OAF3**

1p11	4	6
1q13	0	4
1q18	4	5
1q22	7	0
1q28	6	0
1q38	4	0
1q42	6	0
1q44	7	7
2q13	13	5
2q25	3	4
2q31	0	6
2q42	0	4
2q46	4	0
2q48	4	0
3p11	7	5
3q12	3	0
3q24	0	4
3q26	0	5
3q29	3	0
4p13	3	0
4q19	4	0
4q24	3	0
5p13	3	0
5p15	0	6
6p11	6	0
6q23	10	0
7q11	3	0
7q13	4	0
Xp13	3	0

total	114	66

The number of chromosomal aberrations (chromosomal breaks, chromosomal gaps, chromatid breaks and chromatid gaps) observed in each specimen (OAF1 and OAF3) at each fragile band is provided.

#### (ii) *Chrysochloris asiatica*

A total of 1047 metaphase spreads were analysed from the three specimens included in our investigation: 482 cells from cultures treated with 0.2 μM APC, and 565 from control cultures (table 1). Cells treated with aphidicolin presented the highest number of chromosomal aberrations (68.67% of the total metaphases scored) reflecting a 10-fold increase with respect to the control cultures. A total of 501 chromosomal abnormalities were detected of which chromatid breaks were the most frequent class of aberration encountered in this species (71–94%, table 2).

Analysis of the aphidicolin induced aberrations using the FSM program indicate that chromosomal bands characterised by ≥3 or ≥4 abnormalities per band (depending on the specimen analysed) could be considered fragile. Using these values, a total of 27 fragile sites were detected in the golden mole genome (figure 1b and table 4), with the number of fragile sites ranging from 12 in CAS specimen 1, to 21 in CAS specimen 3 (table 4). Eight of the fragile sites (1p13, 1q13, 1q32, 2p13, 2q22, 2q31, 6q23 and 8q17) were found to be expressed in all three specimens examined (table 4).

**Table 4:** Chromosomal bands that are considered to contain fragile sites in the three golden moles (CAS) specimens studied.

	**No. aberrations**		
**band**	**CAS1**	**CAS2**	**CAS3**

1p15	0	0	8
1p13	8	8	5
1p11	0	0	3
1q13	11	8	5
1q18	3	0	0
1q21	0	8	3
1q32	6	7	4
2p15	0	0	8
2p13	6	6	3
2q17	0	0	3
2q22	7	7	5
2q31	18	26	25
3p11	0	4	0
3q15	0	0	3
3q24	0	0	4
3q25	3	0	0
4q24	0	0	3
5q24	3	0	0
6q23	3	6	6
7q13	0	4	3
7q25	0	0	4
8p15	0	0	6
8q17	5	5	15
9p13	0	0	3
9q14	0	4	6
10q23	0	6	0
12q12	3	0	0

Total	76	99	125

The number of chromosomal aberrations (chromosomal breaks, chromosomal gaps, chromatid breaks and chromatid gaps) observed in each specimen studied (CAS1, CAS2 and CAS3) at each fragile band is provided.

#### (iii) *Elephantulus edwardii*

A total of 1239 metaphase spreads were analysed in the four specimens of this species of which 620 were from cultures treated with 0.2 μM APC, and 619 from control cultures (table 1). We detected 824 chromosomal abnormalities of which chromatid breaks were the most frequent class of aberration scored (67–92%, table 2).

Mirroring the results in the golden mole, the critical value generated by the FSM program for the elephant shrew was ≥3 or, depending on the specimen studied, ≥4 chromosomal abnormalities per band. The number of fragile sites detected ranged from 14 in EED specimen 4 to 20 in EED specimen 3 (table 5); in total 35 sites were considered fragile in the elephant-shrew genome (figure 1c and table 5). Only three of these (2p16, 3q26 and 5q25) are expressed in all four specimens studied (table 5).

**Table 5:** Chromosomal bands which are considered to contain fragile sites in the three elephant-shrew (EED) specimens studied.

	**No. aberrations**			
**Band**	**EED1**	**EED2**	**EED3**	**EED4**

1p22	8	0	0	0
1q11	4	0	0	0
1q15	0	0	5	0
1q16	0	0	3	7
1q21	0	7	16	5
1q31	0	0	8	5
1q33	0	0	16	3
2p16	12	8	7	3
2p13	5	6	0	0
2q13	5	0	3	0
2q26	0	4	3	3
2q31	0	0	3	3
3p26	9	0	4	0
3q26	43	27	11	19
3q28	0	0	17	0
4p11	6	5	0	0
4q11	4	0	0	0
4q18	6	0	5	5
4q24	6	0	0	0
4q26	5	5	0	3
5p12	0	6	4	0
5q25	41	60	18	20
6q16	5	0	0	0
7q12	0	26	4	0
7q14	4	5	0	0
8q11	0	0	0	4
9p12	0	0	0	9
9p11	0	5	3	0
9q14	0	0	0	3
10q12	0	0	5	0
11p12	4	4	0	0
Xp13	5	6	4	0
Xp11	0	6	12	0
Xq13	10	7	0	0
Xq15	0	12	0	0

total	163	159	127	92

The number of chromosomal aberrations (chromosomal breaks, chromosomal gaps, chromatid breaks and chromatid gaps) observed in each specimen (EED1, EED2, EED3 and EED4) at each fragile band is provided.

### Distribution of evolutionary breakpoints and conservation of fragile sites

We plotted all human chromosomal homologies previously described in [53] and [24] onto the ideogram of each of the afrotherian species studied so as to identify bands that delimit human syntenic blocks. Using this approach we identified a set of evolutionary chromosomal bands that correspond to junctions defining human chromosomal syntenies in the three afrotherian species studied herein. These are: (a) Aardvark – 1p11, 1q18, 1q28, 1q35, 2q31, 2q34, 3q29, 4q15, 4q24, 5q21, 6q22 and 6q23, (b) Golden mole – 1q18, 1q21, 2q21, 2q22, 3q28, 4p14, 4q12, 4q22, 6q12, 9q14, 11p13 and 11p12 and (c) Cape rock elephant shrew – 1p26, 1q15, 1q21, 2q17, 2q19, 4q14, 4q24, 5q12, 5q22, 7q12, 10q12, 11p12 and 11q12 (see figure 1). Additionally, we were able to plot the African elephant/aardvark chromosomal syntenies described in [54] that are based on reciprocal painting of these two species thereby providing insights into the association between evolutionary breakpoints and fragile sites among these two species compared to the older, phylogenetically more distant human/Afrotheria evolutionary comparison. Nineteen evolutionary chromosomal bands were detected when comparing the aardvark and African elephant genomes: 1p11, 1q15, 1q18, 1q24, 1q26, 1q28, 1q35, 1q38, 2q13, 2q15, 2q21, 2q25, 2q27, 2q31, 3q12, 3q15, 4p11, 5q14 and 6q23 (figure 1a). On combining these data (human and elephant chromosomal syntenies) 25 distinct evolutionary breakpoints could be defined in the aardvark; of these, six are common to both data sets (1p11, 1q18, 1q28, 1q35, 2q31 and 6q23).

Given these findings, we proceeded to determine if there is a correlation between the position of evolutionary breakpoints and the location of fragile sites in each of the afrotherian species studied using contingency analysis. Of the 12 evolutionary breakpoints identified in the aardvark by reciprocal painting with human painting probes [53], seven are coincidental with regions of fragility as defined by fragile site location (1.8 bands expected if the distribution was random, p = 0.0004) (figure 1a). Of the 19 evolutionary breakpoints identified in the aardvark by reciprocal painting with the African elephant painting probes [54], eight are coincidental with regions of fragility (three bands are expected if the distribution was random, p = 0.0032) (figure 1a). It is noteworthy that of the six bands (1p11, 1q18, 1q28, 1q35, 2q31 and 6q23) that delimit human and elephant chromosomal syntenic blocks in the aardvark genome (see above), all but one (1q35) express fragile sites. Additionally, 27 fragile sites were expressed in the golden mole of which four (1.6 expected, p = 0.07) show correspondence with the 12 evolutionary breakpoints detected by chromosome painting (figure 1b). Of the 35 aphidicolin induced fragile sites in the elephant shrew, six (2.5 expected, p = 0.02) (figure 1c) were coincidental with the 13 evolutionary breakpoints previously identified in this species using human chromosome painting probes [24].

We conducted a more refined analysis of the afrotherian fragile sites by comparing those that are (i) expressed in a single species (i.e., species-specific fragile sites), and (ii) those fragile sites conserved between two or more species (i.e., conserved fragile sites). As above, we assessed each category of fragile site for correspondence with evolutionary breakpoints. Our aim was to test if conserved fragile sites, which are more likely to be ancient fragile sites, might show an enrichment of evolutionary breakpoints. This was borne out by the data which show that of the 12 evolutionary breakpoints identified in the aardvark by reciprocal painting with human painting probes [53], three are coincidental with aardvark-specific fragile sites (1.15 bands are expected if the distribution was random, p = 0.001) and four are coincidental with conserved fragile sites (0.7 bands are expected if the distribution was random, p = 0.001). Similarly, a significant association was found in the elephant shrew (p = 0.03). However, in the case of the golden mole, the tendency was not significant. These data suggest, therefore, that evolutionary breakpoints tend to concentrate more frequently in conserved fragile sites than in those that are species-specific, although only significantly so in two of the three species analysed. Finally, three conserved fragile sites were shared between all three afrotherian species (located in bands 1q28, 1q44 and 3q24 in the aardvark, bands 1q28, 1q32 and 3q24 in the golden mole, and bands 1p22, 1q15 and 1q32 in the elephant shrew). One of these (corresponding to band 1q28 in the aardvark, 1q18 in the golden mole and 1q15 in the elephant shrew) was coincidental with the site of an evolutionary breakpoint in all three species – that corresponding to HSA 2/8, the only chromosomal synteny which strongly supports the Afroinsectiphillia (golden moles, elephant shrews and aardvark) to the exclusion of the elephant [24]).

## Discussion

### Fragile sites and chromosomal evolution

This investigation confirms and extends earlier observations that fragile sites form part of the chromosomal structure in mammals, and that the characteristics underlying their susceptibility to breakage have been conserved during evolution [26,36,37,51,55,56]. Using data from fragile site expression, G-banding analysis, and cross-species chromosome painting, we have identified fragile sites in aardvark, golden mole and elephant-shrew (Afroinsectiphillia) that are located in homologous chromosomal positions in these species. We detected 11 conserved fragile sites in aardvark genome, eight in golden mole, and 10 fragile sites in the elephant-shrew (figure 1). Fragile sites detected in more than one species were regarded as "conserved fragile sites" in order to distinguish them from those that were species-specific.

Although fragile sites have been considered "hot spots" for evolutionary reorganization in a variety of mammalian species (i.e. are regions where chromosomal rearrangements such as fusion/fissions and inversions can repeatedly occur), the data are limited to a single clade, Boreoeutheria [26-28]. This begs the question whether this fragility is a more general phenomenon in mammals, and whether the evolutionary breakpoints that characterise human syntenies detected in the basal Afrotheria similarly correspond at the chromosomal band level with fragile sites detected in other species when using a chemical challenge. Twenty nine fragile sites were detected in aardvark, 27 in golden mole and 35 in the elephant-shrew (Figure 1). A contingency analysis shows that there is a significant association for bands that contain evolutionary breakpoints to accumulate fragile sites in the aardvark (p = 0.0004), as well as in the Cape rock elephant shrew (p = 0.02) genomes. The association was not statistically significant in the golden mole, but there is, nonetheless, a tendency for bands that contain evolutionary breakpoints to accumulate fragile sites in this species (p = 0.07).

The inclusion of the elephant/aardvark chromosome painting data into the analysis offered an opportunity to compare old (aardvark vs. human) and phylogenetically younger evolutionary breakpoints (aardvark vs. elephant) and their correlation with chromosomal fragility. We reasoned that younger breakpoints may show a greater correlation with afrotherian fragile sites than their more ancient counterparts identified in the human vs. afrotherian comparisons. The analysis, however, did not reveal marked differences between the two types of evolutionary breakpoints. When the human chromosomal homologies are plotted against the aardvark genome, seven of the 12 evolutionary breakpoints co-localize with fragile sites (1.8 expected, p = 0.0004). Plotting the elephant chromosomal homologies to the aardvark genome revealed that eight of 19 evolutionary breakpoints co-localize with fragile sites (3 expected, p = 0.0032) indicating that the mechanism causing the fragility is conserved. Given these findings, we then proceeded to determine whether the data inform previous conclusions on chromosomal syntenies thought to underpin the recognition of Afrotheria as one of the four major supraordinal clades of placental mammals (Placentalia).

### Chromosomal signatures in Afrotheria

The recognition of a monophyletic afrotherian clade was initially based on DNA sequence comparisons [13,57] and subsequently supported by the analysis of large concatenations of nuclear and mitochondrial genes [1,2,4,5,16,58,59], unique insertion and deletion events (indels) [8,18,19], comparative cytogenetic studies [23,24,60], morphology [61,62], placentation [63] and, most recently, by whole genome assemblies [11]. Given that Afrotheria is thought to be near the root of the eutherian tree, the organization of their genomes could provide unique insights into the dynamics of mammalian chromosomal evolution.

**Figure 2:**
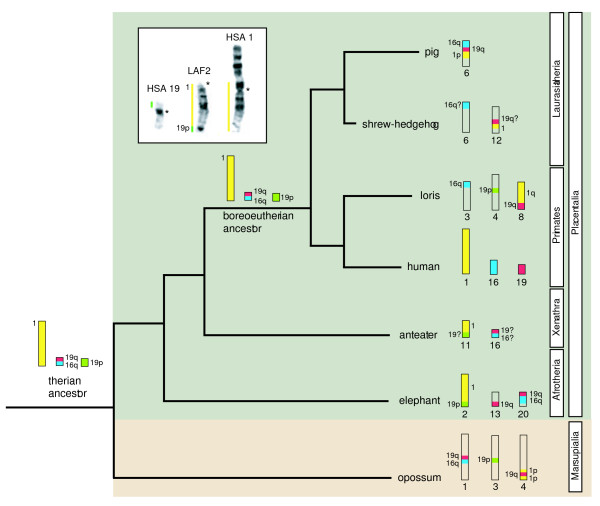
**Phylogenetic tree showing the HSA 1/19 chromosomal syntenies in different mammalian species**. In this scheme we place Afrotheria at the root. There are other competing hypotheses for the basal resolution of Placentalia (see text for details). The ancestral chromosomal forms corresponding to HSA 1, HSA 19p and HSA 19q/16q are represented as single conserved entities in both the therian (Marsupialia + Placentalia) and boreoeutherian ancestors. Data based on chromosomal painting in the African elephant (Afrotheria, [23]), the anteater (Xenarthra, [64, 65]), loris (Primates, [69]), the shrew-hedgehog (Eulipotyphla, [66]) and the pig (Cetartiodactyla, [67]) are represented. The human/opossum homologies are determined from ENSEMBL genome sequence alignments [82]. Question marks indicate instances of ambiguity where reciprocal chromosomal painting has not been performed and therefore unequivocal identification of chromosomal arms is not possible. Inset shows G-banding comparisons between elephant chromosome 2 (LAF 2) and human chromosomes 1 and 19 (HSA 1 and HSA 19); syntenic boundaries are derived from reciprocal chromosome painting data [23]. Note that HSA 1 is inverted to facilitate comparisons with LAF 2. Centromeres are marked by asterisks.

Two human syntenies have been proposed by chromosome painting studies to link afrotherians to the exclusion of other placental mammals (1/19p [23,53] and 3/21/5 [24]). In terms of the former, it is noteworthy that HSA 1/19 has been reported in a xenarthran species (*Tamandua tetradactyla*, [64,65]), the insectivore shrew-hedgehog (*Neotetracus sinensis*, [66]), the pig [67], as well as in the prosimians *Galago moholi, Otolemur crassicaudatus *and *Nycticebus coucang *[68,69], the New World monkeys *Saimiri sciureus *[70] and *Callicebus lugens *[71], and the Old World monkeys *Presbytis cristata *[72], *Pygathrix nemaeus *[73], *Nasalis larvatus *[74], *Trachypithecus francoisi *[74] and *T. phayrei *[74]. However, only three of these studies relied on reciprocal chromosomal painting [23,53,69] with a fourth [67] based on unidirectional painting, but complemented by comparative gene mapping (figure 2). These data are a prerequisite for allowing unequivocal identification of the chromosomal arms (either 19p or 19q) involved in the 1/19 syntenies. Of these, *G. moholi *and *N. coucang *[69] show an HSA 1q/19q association and the pig HSA 1p/19q [67]. In contrast, the African elephant and the aardvark share HSA 1/19p [23,53] begging more detailed analysis of whether 1/19p is truly an afrotherian specific chromosomal signature (figure 2).

**Figure 3:**
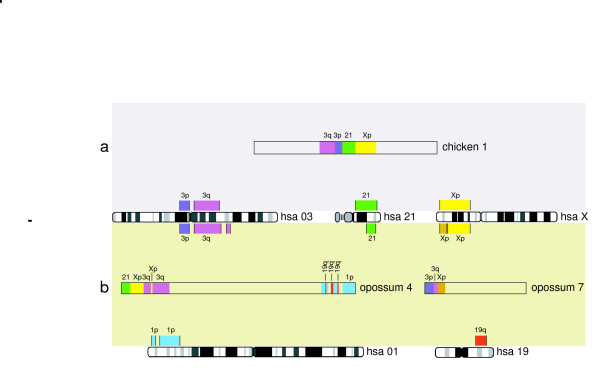
**Representation of the syntenies HSA 3/21/X and HSA 1/19 in the chicken and opossum genomes**. Different colours show contiguous synteny blocks between (a) chicken and human, and (b) opossum and human as determined from ENSEMBL genome sequence alignments [82].

By computationally scanning the genomic assemblies of human and opossum (a marsupial outgroup to Afrotheria and other Placentalia) we sought to validate the HSA 1/19p synteny as a lineage specific, rare genomic change underpinning the monophyly of Afrotheria. In attempting to address this it is important to point out that the defining character in a conserved segmental association is the presence of the breakpoint (i.e. the junctions 1/19p and 3/21/5) since, as has been noted elsewhere [28,60], the size of segments may be altered by subsequent translocations to other regions in the genome, and FISH provides no insight to gene order within the syntenic block which may be altered by intrachromosomal rearrangement. Furthermore, in deciding the most parsimonious pathway to derive a specific chromosomal rearrangement we follow [22] in viewing the independent disruption of a syntenic group to be more likely than the same adjacent synteny being independently reassembled in different lineages. While the opossum genome shows a HSA 1p/19q/1p/19q/1p/19q/1p association on its chromosome 4 (figure 3), this adjacent synteny is different to the HSA 1q/19q found in the prosimian species *G. moholi *and *N. coucang *([69] and figure 2) further reinforcing the finding that HSA 1, reportedly the largest physical unit in the eutherian ancestral genome, has suffered multiple independent fissions [75].

Importantly, however, and of substantial phylogenetic significance, the human chromosomal segment involved in the HSA 1/19p afrotherian synteny is currently ambiguous since the painting data do not allow inference on whether the junction is between HSA 1q or HSA 1p in those species for which there are reciprocal painting data (i.e., elephant and aardvark [23,53]). G-banding homology on the other hand favours HSA 1p/19p (see insert figure 2). If correct, this association would support the monophyly of Afrotheria [23,53] on current information. However, should further analysis reveal its presence in Xenarthra, this would give credence to the recognition of Atlantogenata [76], a clade containing Afrotheria and Xenarthra to the exclusion of Boreoeutheria [11]. Both outcomes underscore the importance of resolving this critical chromosomal synteny for clarifying deep divergences in the eutherian tree although recent strong support for a sister group relationship for Afrotheria and Xenarthra (based on a ~2.2 mega-base data set of protein coding sequences), clearly tips the odds in favour of HSA 1(p?)/19p being a shared synteny for Atlantogenata [12].

Moreover, it is of interest that the human syntenies HSA 1, HSA 16q/19q and HSA 19p have all previously been proposed for the boreoeutherian ancestor [22,28,77] which, if present in the eutherian ancestor, would require a fusion (possibly promoted by an ancient fragile site retained in aardvark OAF 3q29 – figure 1a) to derive the HSA 1/19p synteny that possibly unites Afrotheria to the exclusion of other Placentalia (figure 2). If the same syntenies were present at the therian root all that would be required is a breakpoint in HSA19q with a fusion to HSA1 to give the opossum HSA 1p/19q and HSA1 6q/19q combinations (figure 2).

**Figure 4:**
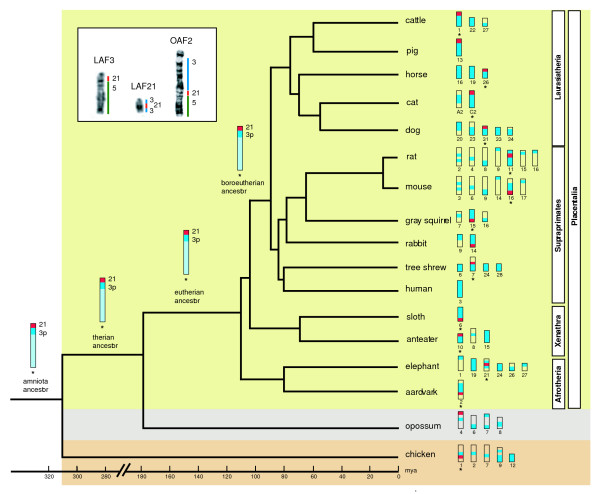
**Phylogenetic tree showing the HSA 3/21 chromosomal syntenies in different mammalian species**. In this scheme we place Afrotheria at the root. There are other competing hypotheses for the basal resolution of Placentalia (see text for details). The ancestral chromosomal forms corresponding to HSA 3/21 are represented as single conserved entities in the amniote, therian, eutherian and boreoeutherian ancestors. Source references for the species shown in the tree are: African elephant [23], domestic pig [84], rabbit [85], tree shrew [86], grey squirrel [87], domestic cat [88], aardvark [53], the xenarthran taxa the amadillo and lesser anteater [64], and the ENSEMBL genome database [82] for the rat, mouse, cattle, dog, opossum and chicken sequence alignments. The asterisks indicate the unequivocal identification of the HSA 3p/21 synteny based on reciprocal chromosome painting or data from the ENSEMBL genome database [82]. The estimated divergence dates follow [11]. The opossum and the chicken are included as representative outgroups species. Inset shows G-banding comparisons between elephant chromosomes 3 (LAF 3) and 21 (LAF 21) and aardvark chromosome 2 (OAF 2) showing the regions of homology to human chromosomes 3, 5 and 21 (HSA 3, HSA 5 and HSA 21); syntenic boundaries are derived from reciprocal chromosome painting data [23].

Using a similar approach we examined the second synteny thought to underpin Afrotheria monophyly, HSA 3/21/5. It was previously argued [24] that the ancestral association HSA 3/21 [53] should be expanded to include segments homologous to human chromosome 5 forming an HSA 3/21/5 segmental combination defining Afrotheria. The authors posit that the most parsimonious explanation for the observed patterns is that HSA 21 appears to have fissioned within Paenungulata; in this regard it is noteworthy that 2q31 is expressed as a fragile site in aardvark (figure 1a), perhaps indicating an ancestral locus. Their reasoning was that the HSA 3/21/5 configuration is present in aardvark, golden mole and elephant-shrew with all three chromosomes retained as intact, conserved entities in the two former species. In the case of the elephant, the fissioning of HSA 21 gave rise to HSA 5/21 (LAF 3) and HSA 1/3/21/3 on LAF 21 (see [23] and inset in figure 4). This rearrangement (the modification of 3/21/5 to HSA 3/21 + HSA 5/21) was recently confirmed [54] in manatee and elephant (data on the hyrax are incomplete) by reciprocal painting with paenungulate species-specific painting probes, and through inferences made from human and aardvark [53]. New information on the Florida manatee (*Trichechus manatus latirostris*, TMA, [78]) resulting from unidirectional painting experiments with human probes similarly show HSA 5/21 on TMA 1, and HSA 2/3/21 on TMA 15.

The widely accepted boreutherian ancestral syntenies (HSA 4/8/4, 7a/16p, 10/12a/22a, 12b/22b, 14/15 and 16q/19q, [22,77]) are all in the opossum genome suggesting their presence in a therian ancestor, and retention for >180 mya (divergence based on stem branches between crown placentals and crown marsupials, [79-81]). One further ancestral synteny (HSA 3/21) deserves special comment, especially with respect to its importance for Afrotheria. Froenicke [22] provided evidence that neighbouring segments homologous to HSA 3 and HSA 21 have been found in all eutherian orders for which there is information, and that the combined analysis of reciprocal chromosome painting data in conjunction with draft genome sequence information for mouse and human showed that the breakpoint is located in HSA 3p, the region closest to the centromere of this chromosome. While there is no evidence of this synteny in opossum, electronic screening of the chicken genome assembly indicates its retention in this species (the HSA 3p segment extends from 76Mb-90 Mb, [82], see figure 3). Opossum has a 21/Xp/3q/Xp/3q synteny in chromosome 4 and, importantly, 3p/3q/Xp in chromosome 7 indicating different breakpoints in Marsupialia (figure 4). In summary therefore, it would seem that HSA 3p/21 was present in the common ancestor of Amniota (i.e. common to turtles, lepidosaurs, crocodilians, birds and mammals) supporting its identification as an ancestral synteny that was present >310 MYA [79], but which was disrupted in the lineage leading to the marsupials. The expansion to include HSA 5 in the afrotherian ancestor is unique [24] and defines the monophyly of this ancient African clade of mammals.

## Conclusion

Using data from fragile site expression, G-banding analysis, and cross-species chromosome painting, we have described a suite of afrotherian common fragile sites that are correlated with evolutionary breakpoints when compared to the human genome. By computationally scanning the newly released opossum and chicken genomes as outgroups to Placentalia, we have shown that the primitive HSA 3p/21 synteny was present in the amniote ancestor, and its expansion to include HSA 5 validates the HSA 3/21/5 synteny as a robust cytogenetic signature that defines the monophyly of Afrotheria. Its fission into two segments (HSA 3p/21 + HSA 5/21) is probably shared by all Paenungulata and may have been facilitated by an ancient fragile site that is still expressed in aardvark. Further, if the human syntenies HSA 1, HSA 16q/19q and HSA 19p (all previously proposed for the boreoeutherian ancestor) were present at the eutherian root, a single fusion (the breakpoint junction being coincidental with a fragile site retained in aardvark at OAF 3q29) would be required to derive the HSA 1/19p synteny that may, with further refined analysis, be found to unite Afrotheria to the exclusion of other Placentalia.

## Methods

### Cell culture and fragile site expression

Fibroblast cell cultures were established from two aardvark (one male and one female), three golden mole (two males and one female) and four elephant-shrew specimens (two females and two males). Twenty four hours before harvesting 50 μl of aphidicolin (APC, 2 mM) dissolved in DMSO was added to cell cultures at a final concentration of 0.2 μM. Concurrent control cultures were established for each experiment. Cells were harvested and chromosomal preparations obtained using standard protocols.

Ideograms were constructed for each of the species according to the standardised karyotype for *O. afer *(2n = 20, OAF [53]), *C. asiatica *(2n = 30, CAS [24]) and *E. edwardii *(2n = 28, EED [24]). (Note that the karyotype presented in [24] was originally incorrectly identified as *E. rupestris*; DNA from the same specimen was subsequently extracted and sequenced, verifying its status as *E. edwardii*; unpublished data). Our banding data show that 177 chromosomal bands define the *O. afer *ideogram, whereas the *C. asiatica *and *E. edwardii *ideograms have 200 and 182 bands, respectively.

All metaphases were sequentially solid-stained and then G-banded to establish the location of the breakpoints. Digital images were captured with a BX60 Olympus microscope utilizing the GENUS imaging System (version 2.75) (Applied Imaging Corporation).

### Analysis of fragile sites

In order to identify which chromosomal bands could be considered fragile regions, a statistical analysis of the distribution of chromosomal abnormalities detected in each specimen analysed was undertaken using the programme FSM (version 995, [83]). In this program, the standardized χ^2 ^and G^2 ^test statistics are used for all chromosomal bands that express non-random breaks or gaps. The hypothesis tested by the programme is that the probability of breakage is equal for all chromosomal bands in a given karyotype using a 0.05 level of significance (α). The FSM analysis gives a critical value for each data set analysed, abbreviated to C_α_, which indicates the lowest frequency of breakage per band that exceeds the level of significance [35,83]. Any chromosomal band with a number of breaks greater than the critical value is considered a fragile site. This value ranged from 3 to 4 depending on the number of chromosomal bands determined in each karyotype (177 bands for the aardvark, 200 bands for the golden mole and 182 bands for the elephant shrew), and the number of breaks detected in each species' data set. In our analyses, chromatid and chromosomal breaks and gaps were treated equally as representing single chromosomal events, and these were mapped to the respective ideogram of each afrotherian species.

### Computational analysis of afrotherian and boreoeutherian lineage-specific syntenies

Data from different studies [24,53,54,78] were used as sources for determining homologies between the human genome and those of the aardvark, golden mole and Cape rock elephant shrew. Evolutionary breakpoints were defined as the limit between each adjacent human homologous region. Evolutionary bands are chromosomal bands that contain evolutionary breakpoints (see [27] for further clarification of evolutionary breakpoints and evolutionary bands). We omitted the centromeres from the analysis and plotted all fragile sites and evolutionary breakpoints onto the ideograms of each of the afrotherian species analysed. A contingency analysis was used (JMP package version 5.1.2; SAS Institute Inc.) to evaluate if evolutionary breakpoints concentrate significantly (p ≤ 0.05) in chromosomal bands containing fragile sites. This is based on the assumption that the chromosomal bands of the genomes of each afrotherian species analysed have the same probability to be affected, independently of the size of the band involved.

The Ensembl genome browser of the Sanger Center and EMBL data base [82] were used for determining homologies between the human genome and those of the opossum and chicken. We used the completed human/chicken (WASHUC 1) and human/opossum (MonDom 4.0) whole-genome sequence assemblies that are available on the Ensembl genome browser [82] to determine syntenic regions between the human genome (NCBI Build 36) and those of opossum and chicken

## Competing interests

The author(s) declares that there are no competing interests.

## Authors' contributions

ARH and TR conceived and designed the experiments. ARH performed the experiments and analysed the fragile site data. Both authors scrutinised evolutionary breakpoint for use in a phylogenetic context, and contributed equally to writing the manuscript.
